# Hypoplastic Left Lobe of Liver with Accessory Caudate Lobe

**DOI:** 10.1155/2013/604513

**Published:** 2013-03-19

**Authors:** Rajani Singh

**Affiliations:** Department of Anatomy, AIIMS, Rishikesh, Uttarakhand 249201, India

## Abstract

During routine dissection, a liver from a cadaver of a female aged 50 years was observed to have hypoplastic left lobe, and on posterior surface an accessory caudate lobe was present to the left of main caudate lobe. It was separated by well-defined fissure from caudate lobe. The fissure for ligamentum venosum was present to the left of accessory caudate lobe. Porta hepatis was present below the new lobe. Prominent papillary process continued with caudate process which in turn is fused with right lobe of the liver. These developmental anomalies of liver may cause confusion during procedures like biopsy, transplantation, and lobectomies. This knowledge may be of immense use to clinicians for the diagnosis and management of hepatic diseases, morphologists and anatomists for new variant, and to embryologists for new developmental defect.

## 1. Introduction

**Figure 1:**
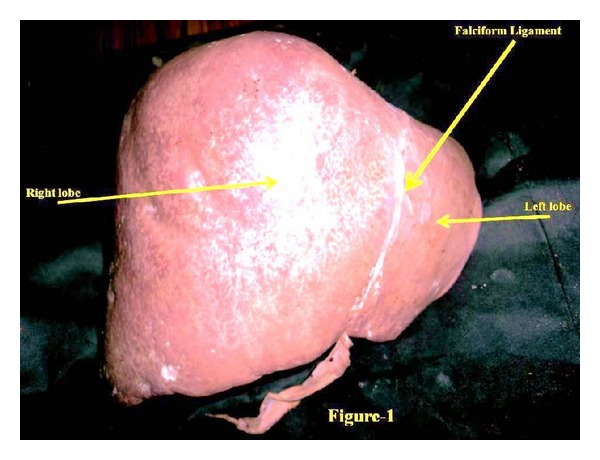
It shows anterosuperior view of liver.

**Figure 2:**
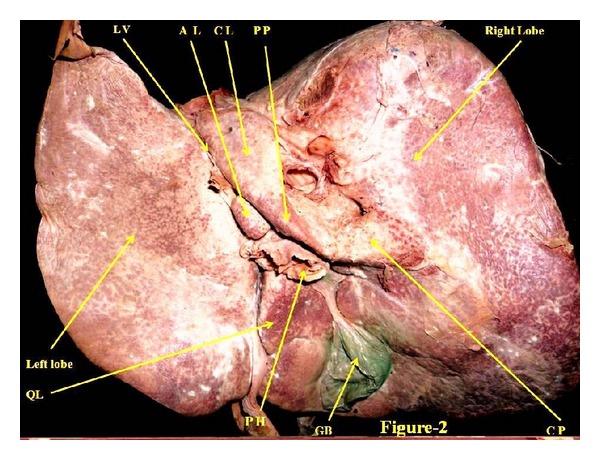
It shows posteroinferior view of liver. CL: caudate lobe AL: accessory caudate lobe, PP: papillary process, CP: caudate process, QL: quadrate lobe, GB: gallbladder, LV: fissure for ligamentum venosum, PH: porta hepatis.

The liver is the largest wedge-shaped gland in the human body. Anatomically it is divided into right and left lobes (Figure 1) based on the attachment of its peritoneal ligaments. The congenital abnormalities of human liver are rare [1], and these are rarer than almost any other organ of the body [2]. Various congenital abnormalities of the liver as agenesis of its lobes, absence of its segments, deformed lobes, decrease in size of lobes, lobar atrophy, hypoplastic lobes, and transposition of the gall bladder and Riedel's lobe have been reported by various authors. It is important to keep in mind the anomalies of liver during the preoperative diagnosis because it will be helpful for the surgeon in planning biliary surgery or a portosystemic anastomosis [1]. Accessory lobe has been described in the vicinity of gallbladder fossa or isolated lobe connected with liver by pedicle or mesentery containing vascular supply. Accessory lobe arising from superior surface [3] and inferior surface [4] has also been reported. The new accessory caudate lobe (Figure 2) is situated in the right of the hypoplastic left lobe and in the left of caudate lobe of the liver under present study. But the configuration, location, shape, and size of the accessory caudate lobe, which is being reported, are altogether different from what has already reported in the literature. These developmental anomalies of liver may cause confusion to clinician during procedures like biopsy, transplantation, and lobectomies. So finding of this new variant under unique configuration of this lobe assumes more importance to anatomists including morphologists, and its knowledge may be of immense use to clinicians in the diagnosis and management of hepatic diseases and to embryologists for new developmental defect. Therefore, it is worth reporting as a new variant.

## 2. Case Report

During routine cadaveric dissection in the Anatomy Department of CSM Medical University, a cadaver of a female aged 50 years was observed having accessory caudate lobe (Figure 2). This lobe was found positioned at inferolateral to the main caudate lobe separated from it by a deep fissure. The fissure for ligamentum venosum was right to the hypoplastic left lobe and to the left of accessory lobe. Porta hepatis was found inferior to the accessory lobe. As far as known this was unique configuration of this accessory caudate lobe.

The dimensions, length, width, and depth of the accessory caudate lobe were 15 mm, 13 mm, and 6 mm, respectively. There was very prominent papillary process (Figure 2) continuing with the caudate process as a border indicating overdevelopment of this part of liver. This unique variant was attached with the liver (Figure 2). These variations of the liver are associated with hypoplastic left lobe of the liver. The observation of diaphragm in cadaver did not show any signs of hernia. Falciform ligament (Figure 1) was attached at its normal site. The position and size of the gall bladder were found normal. No other abnormality was observed in this liver.

## 3. Discussion 

Most of the liver anomalies are congenital. These anomalies cause malformations in the liver. These congenital malformations of the liver include agenesis of the lobes, absence of segments, deformed lobes, decrease in lobe size, atrophy of the lobes, and hypoplastic lobes [5]. Besides these anomalies, multifarious accessory lobes have also been reported by various authors arising from superior and inferior surface of the liver [3, 4]. Accessory caudate lobe observed in the current study was well delineated by fissure from the main caudate lobe and by fissure of ligamentum venosum and porta hepatis and is altogether a new finding not reported so far in the literature. Since the malformations are observed in female cadaver of 50-year age, hence the clinical history is not available. Therefore whether the condition is associated with liver dysfunction or any other clinical condition is not known. The accessory lobe described by various authors is sometimes associated with malformations of other organs like diaphragm and suspensory apparatus of the liver [5]. But in our study, no such malformations were seen. But such accessory lobes may create confusion in interpretation of CT and MRI. 

The embryological basis of the anomalies of liver morphology occurring in the course of organogenesis remains to be elucidated [6]. Dodds et al. gave a hypothesis to explain the formation of caudate liver. According to them during second trimester the ductus venosus rotates rightward as the liver enlarges, so that a small portion of the liver becomes inserted behind the mesentery for the ductus venosus. This part of liver gives rise to caudate lobe of liver [7]. During the formation of caudate lobe, a small portion of caudate lobe may have become separated from it and included in mesentery of ductus venosus to form the accessory lobe. 

Defective development of the left lobe of liver can lead to conditions like gastric volvulus. In the present study left lobe was hypoplastic but it was not associated with either diaphragmatic hernia or gastric volvulus. Postnecrotic cirrhosis, malnutrition, biliary obstruction, and venoocclusive disease have been associated with atrophy or hypoplasia of a hepatic lobe or segment. Since it was observed in a cadaver and history is not available, hence it cannot be said if the hypoplastic left lobe of liver under present study is due to aforementioned conditions. According to some authors the hepatic lobe malformation is not always congenital, and diagnosis of this variant requires evidence of liver dysfunction [8, 9]. It is important to keep in mind these liver anomalies in the correct preoperative diagnosis, because it will be helpful for the surgeon in planning biliary surgery or a portosystemic anastomosis. Whenever there is any such variant of the liver, it is better to examine the other organs as the defective liver could be associated with conditions such as gastric volvulus, diaphragmatic hernia, and portal hypertension. 

Thus knowledge of such variations may be important to anatomists and morphologists for new variant, embryologists for new developmental defect, surgeons for planning surgery involving liver, and imagery specialists for avoiding misinterpretation of CT and MRI.
